# ‘It’s given us the opportunity’: Patient and clinician experiences of serious illness conversations in the NHS acute setting – Results from a UK Qualitative Study

**DOI:** 10.1177/26323524261450637

**Published:** 2026-06-14

**Authors:** Tamsin McGlinchey, Stephen Mason, Jude Robinson, John Edward Ellershaw

**Affiliations:** 1 Palliative Care Unit, University of Liverpool, UK; 2 School of Social and Political Sciences, University of Glasgow, UK

**Keywords:** communication, patient-centred care, qualitative research, outpatients, palliative care, hospitals

## Abstract

**Background:**

Despite legal and ethical commitments to dignified end-of-life care, open communication with seriously ill patients remains inconsistent across the National Health Service (NHS). The Serious Illness Care Programme was implemented in two NHS hospital sites to improve communication and care planning for patients with serious illness.

**Aim:**

Explore the lived experiences of seriously ill patients and their clinicians participating in Serious Illness Conversations.

**Design:**

Qualitative study using a phenomenological approach within an interpretive paradigm. Narrative interviews were undertaken, and Reflexive Thematic Analysis was used to identify patterns of meaning.

**Setting/Participants:**

Outpatient clinics in two NHS hospital sites: a tertiary cancer centre and a renal unit within a city teaching hospital. Fourteen patients (seven cancer, seven renal failure) and nine clinicians (six oncologists, three nephrologists) were interviewed.

**Results:**

The following three interrelated themes were generated from the analysis: (1) Patients described the conversations as ‘*a transformative experience*’, fostering reflection, practical planning, and reframed hope despite a limited prognosis; (2) Serious Illness Conversations were ‘*different conversations*’ from routine consultations, with use of the Serious Illness Conversation Guide enabling deeper, more holistic dialogue; however, they were segregated from routine practice; (3) Systemic and cultural barriers hindered these conversations from becoming embedded, perpetuating inequity of access to these conversations: ‘*Systems, Structure, Process: Serious Illness Conversations, epistemic injustice and cultural violence*’.

**Conclusion:**

Findings from this study directly challenge the commonly held belief that these conversations are inherently negative to reveal the transformative power of these important and necessary conversations. They opened up different types of conversations, giving patients agency to direct certain aspects of their life. However, their integration into routine practice is hindered by structural and cultural constraints. Addressing these barriers is essential to ensure all patients have equitable access to these conversations.

## Plain language summary

Patient and clinician experiences of serious illness conversations in NHS Hospitals in the UK

This study explored how seriously ill patients, and the clinicians who care for them, experienced Serious Illness Conversations in two NHS acute hospitals. Serious Illness Conversations are structured conversations designed to help patients talk about their future, their values, and what matters most to them, to align their care with their wishes. Interviews were undertaken with 14 patients (7 with cancer and 7 with kidney failure) and 9 clinicians (6 oncologists and 3 nephrologists). Patients described the Serious Illness Conversations as transformative. They gave patients space to reflect, plan practically, and reframe hope even when facing a limited prognosis. Patients appreciated clinicians’ willingness to talk openly and honestly about dying and the future. The conversation enhanced their understanding about their illness and enabled them to make decisions about their care and prepare for the future. Clinicians recognised the value of these conversations, which promoted more holistic conversations that went beyond diagnosis and treatment. The Serious Illness Conversation Guide provided a framework for clinicians to structure these conversations with their patients. However, they were seen as different conversations that were organised in addition to routine clinic appointments, making them difficult to integrate into their already busy schedules. Access to Serious Illness Conversations was also unequal. Decisions about which patients were invited were made by clinicians, not patients. Clinicians often faced organisational barriers preventing them from scheduling these conversations such as a lack of available time and space within the hospital setting. This study concluded that Serious Illness Conversations can be powerful and positive for patients and clinicians, but structural and cultural barriers prevent them from becoming routine. Addressing these barriers is essential to ensure all patients have equal access to these conversations.

## Background

The right to a dignified death is enshrined in UK law,^1^ and high-quality, individualised care at the end of life should be a standard expectation.^2,3^ However, considerable variation persists in how end-of-life care is experienced and delivered across the United Kingdom.^4^ Global evidence suggests that a lack of open communication and insufficient involvement of patients and families in care decisions remain major barriers to effective end-of-life care.^5,6^ When timely conversations about treatment and care are absent, it can hinder the delivery of care that reflects individual needs and expectations.^7–9^ A patient’s right to participate in discussions and decisions about their treatment and care is protected within UK professional standards and medical ethics^10^; however, end-of-life decision making can be clinically complex and potentially emotionally charged,^11^ particularly when patients’ and families’ preferences are unclear or misunderstood.^12^ Experiences during the COVID-19 pandemic have reinforced the importance of transparent, empathic communication to support individualised care planning amid clinical uncertainty, especially for those who are seriously ill or dying.^13–15^

Talking about death and dying remains a taboo, despite attempts to ‘normalise’ these conversations.^6,16–18^ In contemporary medical practice, a prevailing culture of cure over care has created uncertainty around how to care for patients where curative options are not a possibility.^19^ Indeed, the recent *Lancet* report on the value of death revealed that although death is an inevitable part of life, it is often treated as if it is not, precluding important conversations about future care and end-of-life planning between patients and clinicians.^6^ Advances in medical interventions have cultivated a belief in the limitless power of medicine to cure, which many argue contributes to a reticence among clinicians to talk about dying, even for patients with serious illness.^20,21^ Evidence shows that end-of-life discussions are not routine practice,^22,23^ despite evidence to suggest that patients want these conversations,^24,25^ and that they are fundamental to developing effective clinical management plans.^23^ In the United Kingdom, the Royal College of Physicians has acknowledged a ‘professional reluctance’ to engage patients and their families in conversations that focus on uncertainty, treatment ceilings, resuscitation status, and death but underline that such conversations are a core responsibility of physicians which must be actively supported within the healthcare system.^23^

The Serious Illness Care Programme is designed to enhance communication, care planning, and quality of life for patients with serious illness by facilitating structured ‘Serious Illness Conversations’ between patients, families, and clinicians.^26^ In the United Kingdom, the programme was piloted as a service improvement initiative in services caring for patients with cancer and end-stage renal disease in two NHS hospital sites. A qualitative study (IRAS: 213686) was conducted alongside the pilot to explore patient and clinician experiences of these conversations in the UK. Data were collected between February 2018 and March 2022, with recruitment paused from March to November 2020 due to the COVID-19 pandemic. A recent integrative review highlighted that serious illness conversations can facilitate proactive discussions that uncover what matters most to patients and help align future care with their values and priorities. However, most studies were from North America, reporting quantitative data from clinical trials, implementation studies, audits of clinical records, or training evaluations, with only a quarter of the included studies reporting qualitative methods on participant experiences.^25^ Although 10 studies stated a specific focus on patient or family perspectives, only three adopted an interpretive qualitative approach using in-depth interviews^27,28^ or structured telephone interviews.^29^ The remaining seven studies used positivist-aligned approaches, reporting data from patient self-completion surveys or structured telephone surveys^30–35^ and a content analysis of conversation transcripts,^36^ which underscores the need for further qualitative research to enhance understanding of patients’ experiences of participating in Serious Illness Conversations. This paper reports the findings from this qualitative study and is the first to include direct patient experience of engaging in serious illness conversations in the UK NHS context.

## Aim

The aim of this study was to explore the lived experience of seriously ill patients and their clinicians of engaging in Serious Illness Conversations in the NHS acute setting.

## Methods

### Epistemological positioning

This study was situated within an interpretivist epistemology, which assumes that reality is produced through individuals’ subjective meanings and that knowledge is co‑constructed through interaction between researcher and participant.^37,38^ A phenomenological orientation informed the study design and interview approach, foregrounding participants’ lived experience and supporting the generation of rich, descriptive narratives.^39^ For analysis, we employed reflexive thematic analysis using a constructionist lens, recognising that meaning is produced through social and contextual processes rather than discovered as an objective truth.^39,40^ This qualitative approach values researcher reflexivity and constructionist meaning-making to produce a nuanced exploration of complex experiential phenomena.^40,41^

### The intervention: The Serious Illness Care Programme UK

The Serious Illness Care Programme facilitates patient-clinician conversations that focus on the patient’s expectations, wishes, and hopes for the future with their illness.^26^ It incorporates systems innovations and clinician training to embed these conversations into the systems and processes of the organisation so that they occur as part of routine care for all patients with serious illness. At the core of the programme is the ‘Serious Illness Conversation Guide’,^42^ providing prompts and structure for clinicians to frame the conversation with their patients.

The Serious Illness Care Programme was implemented across two NHS hospital sites. Clinicians identified patients potentially suitable for a Serious Illness Conversation using the ‘Surprise Question’: ‘Would you be surprised if this patient were to die in the next 12 months?’ Once identified, the clinician introduced the conversation to the patient, provided them with an information sheet, and invited them to a Serious Illness Conversation at their next outpatient clinic appointment. The conversation was not mandatory, and patients were able to decline the invitation. Conversations were intended to be scheduled for a double outpatient clinic slot (30 minutes) to allow adequate time, and patients were welcome to bring a family member or friend. Clinicians documented the discussion using a structured template embedded in the electronic patient record, so that the information was available for other healthcare professionals involved in the patient’s care. A copy was then sent to the patient if they wished.

### Setting

Outpatient clinic services in two NHS hospital sites: One renal unit within a large city centre teaching hospital, and one tertiary cancer centre.

### Population/eligibility criteria

Purposive sampling was used, and participants were eligible for this study if they met the following inclusion criteria:

- Patients:
  ○ Adult patients (>18 years) with a diagnosis of advanced cancer or renal failure attending outpatient clinic appointments within the two study sites
  ○ Participated in a Serious Illness Conversation with their named consultant
  ○ Gave informed consent to take part in the study

- Clinicians:
  ○ Trained in the use of the Serious Illness Conversation Guide within the study sites
  ○ With experience of engaging in at least one Serious Illness Conversation with a patient under their care
  ○ Gave informed consent to take part in the study

### Recruitment

Clinicians were first informed about the study during a 1-day training session on the Serious Illness Conversation Guide, where the researcher (T.M.) obtained verbal agreement to follow up once they began initiating conversations with patients.

Patients were introduced to the study by their named clinician after agreeing to participate in a Serious Illness Conversation and gave verbal consent to be contacted by the researcher (T.M.).

All participants gave their informed consent to take part in the study. The researcher provided potential participants with an information sheet about the study and invited them to take part in a one-to-one interview. Potential participants were encouraged to ask any questions about the research study and the research team, and contact details were provided on the information sheet. For those who agreed, a suitable date, time, and location were arranged, and written consent was obtained before the interview commenced.

### Sample size

A maximum of 20 patients and a maximum of 10 clinicians was the target sample size for this study. This decision was taken using Malterud et al.’s^43^ concept of ‘information power’, that the more information a sample holds (as ‘informants’), the need for fewer participants, and the advice from Sim et al.^44^ that justification regarding sample size should necessarily be pragmatic.

Due to the novel approach of the Serious Illness Care Programme within the two implementation sites, the number of eligible key ‘informants’ was likely to be lower than recruiting participants identified from outpatient clinics regarding a more ‘generic’ experience of healthcare delivery. For example, clinician eligibility is necessarily bounded by the number of those trained for implementation of the Programme, and patients by their identification for, and agreement to participate in, a Serious Illness Conversation. As a result, this group of participants holds significant ‘information power’ with which to anchor the analysis and findings from this study.

### Data collection

One-to-one narrative style interviews were undertaken by the researcher (T.M.) using an informal, conversational style,^45,46^ designed to be naturalistic and retain the ‘messiness’ of real-life dialogue.^47^ Interviews were either face-to-face or, due to restrictions following the COVID-19 pandemic, by telephone. This was a pragmatic adaptation that enabled the study to continue, with remote interviewing recognised as a valid alternative to in-person interviewing.^48^ A topic guide was developed for both patient and clinician interviews, incorporating a context-setting open question and optional prompts to support the flow of conversation, to elicit the lived experience of patients and clinicians of participating in these conversations. Participants were encouraged to talk about what was important to them, with the researcher acting as an ‘interested learner’.^49^ The topic guides used for this study have been included as a Supplemental File (S1: Appendix 1). Interviews were audio-recorded and transcribed verbatim for analysis. All interviews were audio recorded and transcribed verbatim. Transcripts were anonymised prior to analysis by removing identifiable details such as names, locations, and protected characteristics.

## Analysis

Reflexive Thematic Analysis^50,51^ was used to identify patterns of meaning related to participants’ experiences of engaging in these conversations. Situated within a constructionist paradigm, where participants’ lived experience was analysed within the ontological perspective that individual meaning is socially constructed through meanings and practices, the analysis sought to stay close to the data and participants’ language, in order to explore the underlying ideas, assumptions, and concepts shaping their accounts.^41^ T.M. read and reread the transcripts to become familiar with each narrative, recording written reflections to capture initial impressions of individual stories. T.M. manually coded the data using open, inductive coding, allowing the analysis to reflect both participants’ constructed meanings and the researcher’s interpretive lens.^41^ Themes were then developed around central concepts that captured patterns of shared meaning across the dataset, forming a coherent, meaning-based interpretive narrative.^51^

### Reflexivity

The researcher for this study (T.M.) undertook both the data collection and the analysis. At the time of the study, T.M. was a research assistant in palliative and end-of-life care, conducting this research for a PhD. T.M. has worked as a researcher in palliative and end-of-life care for over 10 years, with experience in conducting research studies that explore sensitive topics. Whilst experience brings with it an increased understanding of the clinical context within which the study is being conducted, it could also be argued that it increases the risk of ‘taken for granted’ interpretations.^49,52^ Importantly, the analytic process was supported by engagement with reflexive practices,^50^ such as through supervisory discussions with senior academics on the study (S.M., J.E., and J.R.), systematic revisiting of coding based on developing understanding, and presentation of developing themes to clinical audiences. These processes supported the researcher (T.M.), leading to heightened engagement with the data, and were important to mitigate the risks of analytical foreclosure^53^ and potentiate interpretations that remained grounded in the participants’ accounts rather than shaped by latent assumptions of the researcher.

This manuscript has been written in accordance with the COnsolidated Criteria for Reporting Qualitative (COREQ) research checklist (see S2: Supplemental Table for the COREQ checklist).

## Findings

### Participants

Sixty patients in total participated in a Serious Illness Conversation with their clinician during the data collection: 45 with a diagnosis of cancer, 15 with a diagnosis of renal failure. Sixteen patients with cancer, and seven patients with renal failure, were contacted to take part in a narrative interview. Of the 16 patients with a cancer diagnosis, 3 patients died before contact was made, and 6 declined to take part in an interview. Seven patients with cancer took part in this study. All seven patients with renal failure were recruited. Overall, 50% of patient participants were female, and 57% were aged between 60 and 90 years old. All patients were of white British ethnicity.

Ten oncology consultants and three nephrology consultants were trained as part of the Serious Illness Care Programme and were contacted to take part in a narrative interview. Four oncology clinicians declined to take part in the study. Six oncology consultants and all three nephrology consultants took part in this study.

Tables 1 and 2 outline the 14 patient and 9 clinician participants that took part in this study. Some of the patient participants chose to have a close family member in attendance, and this has also been included for contextual information. All interviews with patients were conducted within 4 weeks of the conversation, and for clinicians within 4 weeks of their first conversation with a patient.

**Table 1.** Patient participants: demographic information, diagnosis, and interview context.

Patients	Interview mode	Gender	Family member present?	Age range	Cancer diagnosis/renal failure
P001	Face to face	Male	Yes – wife present	50–60	Cancer
P002	Face to face	Female	No	40–50	Cancer
P003	Face to face	Male	Yes – wife present	70–80	Cancer
P004	Face to face	Male	Yes – wife present	70–80	Cancer
P005	Face to face	Female	No	50–60	Cancer
P006	Face to face	Female	No	50–60	Cancer
P010	Face to face	Male	No	80–90	Cancer
PR001	Face to face	Male	No	60–70	Renal failure
PR002	Telephone	Female	No	80–90	Renal failure
PR003	Telephone	Female	No	70–80	Renal failure
PR004	Telephone	Male	No	50–60	Renal failure
PR005	Telephone	Female	No	50–60	Renal failure
PR006	Telephone	Male	Yes – daughter present	60–70	Renal failure
PR007	Telephone	Female	No	70–80	Renal failure

**Table 2.** Clinician participants: Demographic information, and interview context.

Clinicians	Interview mode	Gender	Ethnicity	Oncologist/Nephrologist
OC001	Face to face	Male	White British	Oncologist
OC002	Face to face	Female	White British	Oncologist
OC003	Face to face	Female	White British	Oncologist
OC004	Face to face	Male	White British	Oncologist
OC005	Face to face	Female	Any other white background	Oncologist
OC006	Face to face	Female	Asian British	Oncologist
RC001	Telephone	Female	White British	Nephrologist
RC002	Telephone	Female	Asian British	Nephrologist
RC003	Telephone	Female	White British	Nephrologist

### Qualitative interviews

**Figure 1.**
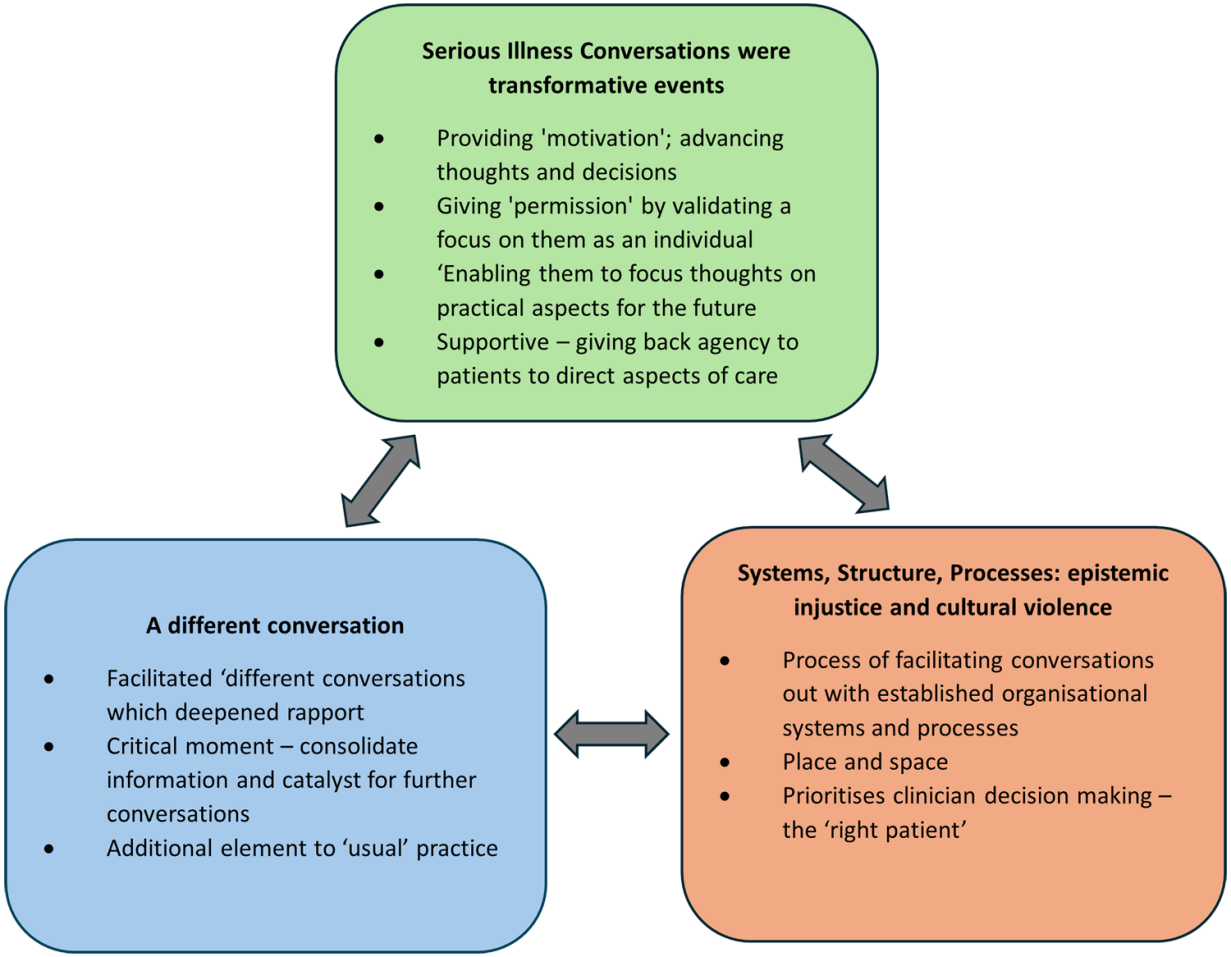
Three interrelated themes generated from the analysis: Serious Illness Conversations were transformative events; A different conversation; Systems, Structure, Process: epistemic injustice and cultural violence.

Findings are presented under the following three interrelated themes (Figure 1): firstly, patient narratives highlighted that despite the emotional load, ‘*the Serious Illness Conversations are a transformative experience*’ which brought about observable gains for patients, such as providing motivation, validating a focus on them as an individual, and enabling them to focus on practical aspects for the future. Secondly, patient and clinician narratives revealed they manifested as ‘a *different conversation*’ to those had in routine clinic consultations; however, despite the positive experiences they brought, they were seen as additional to routine care rather than an integrated part; thirdly, ‘*Systems, Structure, Process: Serious Illness Conversations, epistemic injustice and cultural violence*’ draws on clinician narratives to highlight the influence of organisational culture, systems, and processes which can restrict, inhibit, or prohibit Serious Illness Conversations in practice, drawing on concepts of ‘epistemic injustice’^54^ for patients (privileging clinician testimonies and silencing those of patients, preventing them from being heard and understood), and ‘cultural violence’^55^ for clinicians (hidden institutional organisational systems and structures that influence their ability to provide the care they wish to provide for their patients).

### Serious illness conversations are a transformative experience

For patients in this study, participation in the Serious Illness Conversation brought many benefits. They were motivating conversations that led to new insights into their health situation, helping to restore or reframe a hope for their future despite a limited prognosis. The conversation was a reflective experience for patients that did not exacerbate concerns but focused discussion on them so that tangible ‘action’ could be taken.

yes, it was a bolt out of the blue yes, I’ve had cancer for 17 years erm . . . and erm, I’ve dodged a bullet. I’ve had radiotherapy, chemotherapy, I’m now back on chemotherapy erm but now [my consultant] has kind of put a . . . a semi date on it [end of life], which is 12 to 18 months, erm, which has given us the opportunity to do this sort of thing [Serious Illness Conversation] which is to go through where we want to be and what we want to happen. (PC003, male, 60–70, cancer)

That is not to say that all patients described being ‘accepting’ of their diagnosis, prognosis, or their perceived expectations of what their future holds. For some patients, there was a discernible sense of fear and worry that was still present following the conversation. However, what the conversation did was get them to open up and express their concerns in a way that enabled direct acknowledgement of those worries so that they could make meaningful plans to manage or mitigate against them. Patients appreciated clinicians’ willingness to talk about dying and the future, which brought the ‘reality’ of their illness to the forefront.

I think some of the questions, yeah, you ask that kinda thing yourself, but I think it is useful for your oncologist to bring those questions up. I think the thing is, I mean your oncologist is kinda sussing you out all the time, and they get to know, I think, err how much you do want to know, and what the next things are because nobody knows what’s gonna happen, so there are, there’s like if’s and buts isn’t there . . . but it does kinda confirm other things. Cos like I say, it did prompt me . . . cos I was saying because I do, erm, [my consultant] knows this, I do push it. I go for my appointments, and I just push it [away] and get on with everything else. (PC005, female, 50–60, cancer)

Many patients, however, described their understanding of illness in terms of a dual awareness: the ‘realistic’ expectation that they would ultimately die from their disease, alongside the enduring hope for more time. Patient accounts reinforce that facing death is a complex process, and that stages of illness at different points will involve components of denial and acceptance. What patients valued from the conversation was the sense of agency they gained over decisions about their care, and how best to manage their illness.

. . . I know people can come to terms with things . . . but really the actual dying part . . . I don’t really want to think about that yet. I’m not at that point. So, it can be kinda blinkered kinda view maybe, maybe other people would but I don’t. That’s not in my future at the moment . . . no . . . no . . . But I do know, I mean, don’t get me wrong, I do understand, and I do feel that preparing yourself mentally is a good thing. Bits at a time . . . you know when you talk about your prognosis and what treatments are available and how you cope and all that, it kinda just brings it all in smaller steps which is cope-able with. But also I do think at some point you need to kind of think to yourself, well actually, this might happen, or I might need to get this sorted. Not that I am, but . . . I will. (PC005, female, 50–60, cancer)

For other patients, having the conversation enabled them to focus their thoughts on practical aspects for the future; the tangible things that they could plan for or do to lessen worries or concerns for themselves and their families. For example, enabling patients to make plans to ensure that loved ones are taken care of if they become unable, or share new information with loved ones so that they are able to take better care of them.

I don’t want to go in a home. That’s definitely what I’ve said to [my son]. So we did say that if I got any ill, y’know, worse, and I’m not able to look after myself, we’d get like a home help in and things like that. Cos I still wanted to have my independence. I think the independence part is the important part. I think that would be hard. If I didn’t have my independence, what’s the point? . . . I hadn’t thought of that part of it before until [the Dr] brought it up [in the Serious Illness Conversation]. (PR003, female, 70–80, renal failure)

[the conversation] has sort of given us the opening, erm . . . [my husband] felt relaxed and able to just say [what he wanted for the future] . . . and I said well what do you want to do then? He said well, I’d sell the house, he said, and move to the country. Ok. He said would you mind that? I said, no, do what you like. Be happy, you know. So, it, it’s opened things up which I think we needed rather than like, you know, no, talk about that later, we’ll talk about that later, talk about that later. (PC002, female, 40–50, cancer)

### A different conversation

Analysis revealed that the scheduled Serious Illness Conversation prompted ‘different’ kinds of conversations between patients and clinicians that had not occurred in previous routine outpatient clinic appointments. For patients, the conversation was a significant event that opened up deeper conversations with their clinician, providing an opportunity to consolidate information from previous clinic appointments or discussions with family or friends.

I think, in a way, she, I think there kinda comes a point in your treatment, so we have touched on things, and, questions have been answered that I’ve asked, and things like that. How is this different? It’s different in the fact that it all came together in one kind of, session, if you like. So, it covered quite a lot of information and sometimes as well you forget what’s been said previously, or you just push it to the back of your mind, so it was, I did find it very useful, it’s kind of confirming things that I already thought I knew, and also seeing what was next down the line. Yeah, that kind of thing. (P005, female, 50–60, cancer)

For clinicians, there was an underlying belief that the core function of a doctor is to diagnose and treat illness and that their caring role was defined by their ability to provide treatment. The Serious Illness Conversation enabled clinicians to reframe their caring role within this ‘different’ clinic consultation within a broader understanding of patient care that encompasses more than just ‘active’ treatment. The conversation acted as a catalyst, opening up opportunities for different and ongoing conversations that centred on the holistic needs of the patient.

Often there’s the sort of really, almost like falling off a cliff between, we’re doing active treatment and suddenly we’re not doing active treatment, so what happens next . . . [the Serious Illness Conversation means you can] . . . have a conversation and a talk about that transition. (OC001, male, oncologist)

the conversations that I have had . . . erm . . . I found them to be useful and the patients, I think, they seemed to have really enjoyed the conversations and that opportunity to talk about things we wouldn’t necessarily always talk about. (OC003, female, oncologist)

However, in practice, these conversations were marked out as ‘different’ and segregated from routine clinic consultations. They were perceived by clinicians as ‘additional’ to, rather than an anticipated and integrated aspect of care delivery. Clinicians frequently situated this perception within the pressures of busy outpatient clinics that prioritised adherence to the usual patient pathway, with little room for ‘diversion’ or ‘adaptation’.

it’s erm a reality that initially it’s an additional time slot that’s probably longer than a time slot I would otherwise have so it feels like new activity in a service that’s already bursting at the seams. (OC004, Oncologist, male)

### Systems, structure, process: Serious Illness Conversations, epistemic injustice, and cultural violence

Clinician accounts revealed inequities in access to Serious Illness Conversations, with eligibility ultimately determined by clinicians rather than patients themselves. While some clinicians drew on prior discussions in which patients had raised questions or concerns about the future, the decision of if, when, and how patients were invited to a Serious Illness Conversation was made independently, without direct involvement or collaboration with the patient; for example, as one nephrology consultant responded: ‘*I’ve chosen patients*’ (RC001, female, nephrology consultant). This places the clinician in an epistemically privileged position, in which their expert clinical judgement is prioritised, excluding or silencing the patient's voice within clinical communication (epistemic injustice). For example, one renal patient reflected this potential by highlighting the frustration they experience if they think that they are not being ‘listened to’, emphasising the real need for clinicians to offer patients opportunities to engage in meaningful conversations about their care.

But if people [clinicians] listen, I mean, it wouldn’t be so bad if people listened, but . . . they’re a couple of things that could be made [better], if you’re on about our time and the whole welfare, they could be things that could and should be looked at. (PR001, male, 50–60, renal failure)

Whilst clinicians described being able to clinically identify patients who may benefit from a Serious Illness Conversation, this was not routinely disclosed or discussed with those patients, excluding them from being an equal partner in the decision about when and how they engage in such conversations.

I do recognise that particularly within our older, heavily co-morbid dialysis population that sort of a lot of the conversations with regard to expectations and end of life planning perhaps happens at quite a late stage, and that, erm, although as clinicians often we can sort of see that coming, it isn’t something that’s sort of openly discussed, currently. (RC002, female, nephrology consultant)

When conversations were initiated, despite some initial apprehension, clinician stories highlighted the positive role that the Serious Illness Conversation Guide played in facilitating these conversations with their patients during the clinical consultation. Using the Guide gave clinicians a sense of permission and increased confidence to begin these conversations, providing them with a framework that supported them with language and structure that gave voice to the patients and allowed their priorities, concerns, worries, and hopes for the future to be discussed in a way that was more individualised and meaningful.

I think the Serious Illness, the way the questions are phrased, it does allow someone to lead, does allow the patient to lead with the answers and what they want to talk about, which I think is a good aspect of the guide, rather than being kind of led by, we’ve got to make decisions about certain things. And I hope, I felt that the way the conversation went allowed the patients, people, to actually talk about what they wanted to talk about, rather than necessarily what I wanted to talk about. (RC01, female, nephrology consultant)

However, clinician accounts revealed that inflexible institutional systems and structures in the hospital exacerbated inequity of access for patients, and inhibited clinicians from facilitating them, leaving clinicians unable to deliver the care they wished to provide (cultural violence). Clinicians came up against embedded organisational systems that were not flexible enough to allow them to hold serious illness conversations for all patients who may benefit, despite their best attempts. For example, they described needing to appropriate scarce office space or other physical spaces in the hospital just to be able to hold the conversation in an appropriate setting, or they were left to carve out time themselves in their already busy outpatient clinic schedules to ensure time was allocated for the conversation. In many instances, clinicians described being unable to schedule conversations at all due to the lack of available clinic space and time.

clinic space is a premium here, most people can’t get access . . .’cos at the moment as I say we are adding on to the existing clinics. I think we feel perhaps a little bit unsupported doing it in the clinics peripherally because we don’t have, there’s varying levels of clinic support. (OC003, female, Oncology Consultant)

Sometimes finding space is often quite limited; finding sort of a quiet private area around the dialysis unit or somewhere else in the hospital is often a challenge. (RC002, female, nephrology consultant)

Systems of care should not rely solely on the abilities of individual consultants to ensure that necessary care can be delivered to patients who need it, when they need it. This sentiment is encapsulated in just a few words by one oncologist, who became quite emotional while talking about the challenges they faced, and how unsupported they felt in trying to facilitate these conversations.

[you need] to make sure the infrastructure actually gets organised because I can’t do that, and I really can’t you know I haven’t got the time. (OC005, female, oncologist)

Despite some ‘work arounds’ that were able to be made, it was not always possible for clinicians to guarantee additional time for the appointment, and when this happened, it impacted their perceived ability to have the conversation ‘appropriately’ in the short time they had available in the usual clinic slot. In this situation, clinicians often choose either to delay the conversation, or not to have the conversation at all, rather than compromise the ‘quality’ of the conversation by trying to fit it into the limited time available.

The problems I’ve come up with is more to do with the time constraints and me sort of . . . being able to within that time, bring it in an appropriate manner and . . . and if I didn’t feel it would be appropriate, I wouldn’t, if I’m being honest. (OC006, female, oncologist)

## Discussion

### Main findings

Patient and clinician stories illustrated tangible and existential benefits from engaging in Serious Illness Conversations. For patients, the conversation created the possibility and opportunity to be more equal partners in discussions about their care, catalysing positive decisions or shifts in thinking about the future with their illness, reframing hope for the future in light of a limited prognosis, and enabling care to be aligned with their values and preferences. Through the conversation, clinicians gained a deeper understanding of the patient’s individual needs beyond their physical and clinical needs. However, clinicians were gatekeepers, deciding which patients were invited to a conversation and when. Their accounts highlighted a reticence to engage patients where it appeared they were less ‘accepting’ of their clinical situation, potentially marginalising patients who do not conform to the more palatable ‘accepting patient’ and excluding them from decisions about their care.

Findings also revealed that Serious Illness Conversations were viewed as ‘different’ conversations organised in addition to the routine clinic consultations that clinicians have with their patients. Despite positive benefits, this ‘difference’ resulted in a segregation of the conversations from routine care, making it difficult to make room for them in the existing hospital system. Clinicians faced consistent barriers to embedding this process into existing hospital structures. Despite their best efforts, it was not possible for clinicians to guarantee that the conversation could happen for all patients. The onus was on the clinicians themselves to carve out the time and create the physical space for the conversations, jeopardising the continuity of these conversations as part of routine practice.

### What this study adds

For patients in this study, Serious Illness Conversations were ultimately meaningful and important, even when accompanied by fears and worries about the future. Although such discussions carried an emotional weight, drawing on Lazarus and Folkman’s Transactional Model of Stress,^56^ any immediate ‘situational upset’ that patients experienced did not translate into prolonged or unnecessary distress. Patient narratives from this study challenge widely held societal assumptions that conversations about future care planning and the end of life are inherently negative, as well as health professional assumptions that these conversations should only occur once a patient is deemed accepting of their situation. Instead, they support Zimmerman and Rodin’s view that while these conversations may be naturally upsetting, they should not be avoided for fear of causing undue distress.^57^ Indeed, Geerse et al.^36^ in their content analysis of serious illness conversations, found that clinicians often struggled when patients used emotional language during the conversation, opting to instead shift the focus to medical discussions about treatment options. Importantly, this study found that the conversations generated positive benefits for patients that extended beyond the clinical consultation, underscoring their value despite the emotional load. Indeed, these findings explicate those of Borregaard Myrhøj et al.,^27^ that these conversations created space for patients to address existential concerns and strengthen a shared understanding that can transform communication between patients and clinicians. Findings from this study should reassure clinicians that, although potentially challenging, such discussions do not negatively affect the patient-clinician relationship, explicating insights from telephone surveys by Kumar et al.^35^ and King et al.,^58^ or unduly increase patients’ emotional burden, which Paladino et al.^59^ have previously identified as a major barrier to clinician willingness to initiate them.

Previous research by Morberg Jämterud and Sandgren has argued that clinicians struggle to initiate Serious Illness Conversations due to existential discomfort with death and ethical concerns about timing, honesty, and harm.^60^ Findings from this study build on this by underlining how the inherent uncertainties of predicting prognosis and the responsibility of identifying when a patient may benefit from a Serious Illness Conversation exposed clinicians to what Elton terms ‘epistemic risk’, or fear of ‘getting it wrong’.^20^ Clinicians feared inviting the ‘wrong patient’ to a conversation, resulting in unnecessary distress for the patient. This fostered a reluctance to engage patients unless they were confident that the patient was accepting of their situation and, therefore, more likely to be receptive to the conversation. However, patient narratives from this study reinforce that acceptance and denial are not mutually exclusive, echoing arguments from Zimmerman and Rodin^57^ and Zimmerman^61,62^ suggesting the ‘accepting patient’ is an illusion. Patient accounts highlight that meaningful communication about serious illness and death must acknowledge and work with both states of being. However, conversations need not explicitly focus on death or the prospect of death to be valuable, and patients in this study gained most from the conversation when it facilitated discussion about how to minimise their suffering and optimise the management of their illness for the time they have left.

In this study, clinicians acted as gatekeepers to the Serious Illness Conversations, determining not only which patients were invited but also when such conversations were deemed appropriate, reflecting entrenched epistemic hierarchies in medical institutions where clinicians’ biomedical expertise is often privileged over patients’ experiential knowledge. Clinicians often described inviting patients to a conversation only when they appeared ‘accepting’, or when time and space allowed. Drawing on Fricker’s notion of epistemic injustice, such gatekeeping can perpetuate inequality in access to these important conversations, reinforcing the silencing of patients’ voices and limiting their ability to express concerns or preferences for their care in ways that are recognised as legitimate.^54^ These findings suggest that without deliberate efforts to optimise access to Serious Illness Conversations, patients risk being excluded from important conversations and decisions about their care, undermining the patient-centred approach that the intervention is meant to promote. Further supporting this, Ng et al.^63^ identified that emotional and cognitive barriers can impede patients’ readiness for serious illness conversations unless clinicians adopt active, supportive strategies to engage them, such as feeling overwhelmed or confused about their illness, withdrawing, or avoiding discussions and focusing on the ‘worst case scenario’, demonstrating that readiness is not something a patient must already have, but something that can develop when clinicians and patients work together intentionally. These findings suggest that patients should be actively involved in determining when Serious Illness Conversations occur, with timing negotiated collaboratively rather than determined unilaterally, and through ongoing conversations that evolve based on patients’ needs. Sellars et al.,^64^ in their study of patient and caregiver perspectives on advance care planning in end-stage renal disease, specifically highlighted the disappointment and anger that can result if patients feel they have been excluded from important conversations, or where they feel information has been withheld, about their care and treatment. This also aligns with Carel and Kidd’s critique of how patient testimonies are frequently devalued in healthcare^65,66^ and supports Fischer and Ereaut’s argument that the current dominant model of clinician-led clinical communication often reinforces clinician authority at the expense of patient empowerment.^67^

Our findings reinforced those of Swiderski et al.^68^ to reveal that clinicians’ ability to facilitate serious illness conversations for their patients was significantly hindered by the lack of supportive infrastructure, such as time constraints and the absence of workflow integration within outpatient clinics. These systemic limitations, while not overtly harmful, reflect what Galtung describes as Cultural Violence: the normalisation of institutional barriers that obscure and perpetuate harm.^55^ Clinician accounts suggest these conversations currently exist outside the boundaries of routine NHS care and are perceived as an ‘additional’ rather than an integral part of clinical practice. As a result, clinicians had to adapt, circumvent, or bypass the established process to secure time and space for these discussions in their outpatient clinics; efforts that were not always feasible for every patient, causing clinicians concern and frustration, leaving them feeling powerless to affect any change, and exacerbating moral distress.^69^ Findings from this study extend those of Paladino et al.^70^ and Ng et al.,^63^ demonstrating that obstacles to initiating Serious Illness Conversations are not solely due to individual clinician discomfort or reluctance, or patient readiness for the conversation. Instead, they are compounded by embedded systemic constraints at the organisational level, which create persistent barriers to these conversations, reinforcing inequity of access. Lagrotteria et al.^71^ found that implementing the Serious Illness Care Programme promoted positive behaviour change in clinicians, by broadening ‘goals-of-care discussions’ beyond code status to more meaningful, person-centred conversations about patients’ values, goals, and preferences, enabling them to deliver the ‘humanised’ care they strived for. However, such changes cannot be sustained without a change in organisational culture to one that supports and prioritises these conversations as a routine part of healthcare practice.

### Strengths and limitations

It is important to consider some limitations to contextualise these findings. This study has a small sample with 14 patients, and 9 clinicians, therefore, claims of representation would be overstated. However, the aim was to explore the individual experiences of participants, to illuminate the complexities of these conversations within the specific context of this research study in the UK NHS setting, rather than generalise these findings to the wider population. At the time of writing, this is the first qualitative study in the United Kingdom to interview patients about their individual experiences of participating in a Serious Illness Conversation with their clinicians in the NHS context. Reflecting on the findings, it may have been pertinent to include a longitudinal element within the study design, potentially providing valuable insights into any changes in thoughts or behaviour due to the conversation, including how and when these conversations are revisited and the impact on communication and care planning for patients and clinicians in the long term.

### Implications for clinical practice and research

Epistemic hierarchies and restrictive, inflexible, organisational systems in the NHS hospital environment were identified as barriers to embedding Serious Illness Conversations into routine practice. This suggests a need for further research into how individual and systemic influences interact to shape these conversations. Finding ways to empower and enhance both patients’ and clinicians’ readiness for these conversations is essential to ensure equity of access and continuity of these conversations for all patients who want them. These findings also reflect wider societal fears and discomfort with talking about death and dying, revealing a tension between denial and acceptance in both clinician and patient narratives. At the same time, they resonate with Zimmerman’s argument^62^ that a lack of acceptance does not necessarily preclude the ability to provide effective and meaningful end-of-life care. This suggests a need for further research to explore how Serious Illness Conversations unfold, particularly in relation to the emotional and existential impacts of these conversations, and how patients draw on acceptance and denial to frame and navigate these discussions with their clinicians.

### Conclusion

This study offers important new insights into the experience of Serious Illness Conversations in the UK NHS context, through the direct involvement of patients and clinicians. These findings highlight the transformative potential of these conversations for patients, challenging assumptions that these conversations are inherently negative. They provided a unique opportunity for meaningful conversations with their clinicians about their future and care that had not been possible in previous routine clinic appointments. The Serious Illness Conversation Guide provided clinicians with a tangible framework to open up these conversations with their patients. However, findings from clinicians in this study also illuminated systemic barriers within UK NHS hospital settings that hinder their routine implementation, including epistemic hierarchies that perpetuate inequity of access to these conversations. Without proactive, open, and honest clinical communication about care, patients may end up receiving care that is not aligned with their values and priorities. This increases the potential for avoidable harm and undermines the NHS’s commitment to ensuring both clinical staff and patients are empowered to contribute to safer care.^72^ Just as the quality of end-of-life care is argued to be a reflection of how we treat all vulnerable people,^73^ the way clinicians engage in serious illness communication should be viewed as an indicator for all patient-centred care across the healthcare system. Therefore, supporting patients and clinicians to engage in Serious Illness Conversations should be recognised as a key imperative for the NHS, to ensure all patients have equitable access to these important conversations.

## Supplemental Material

sj-docx-1-pcr-10.1177_26323524261450637 – Supplemental material for ‘It’s given us the opportunity’: Patient and clinician experiences of serious illness conversations in the NHS acute setting – Results from a UK Qualitative Study Supplemental material, sj-docx-1-pcr-10.1177_26323524261450637 for ‘It’s given us the opportunity’: Patient and clinician experiences of serious illness conversations in the NHS acute setting – Results from a UK Qualitative Study by Tamsin McGlinchey, Stephen Mason, Jude Robinson and John Edward Ellershaw in Palliative Care and Social Practice

sj-docx-2-pcr-10.1177_26323524261450637 – Supplemental material for ‘It’s given us the opportunity’: Patient and clinician experiences of serious illness conversations in the NHS acute setting – Results from a UK Qualitative Study Supplemental material, sj-docx-2-pcr-10.1177_26323524261450637 for ‘It’s given us the opportunity’: Patient and clinician experiences of serious illness conversations in the NHS acute setting – Results from a UK Qualitative Study by Tamsin McGlinchey, Stephen Mason, Jude Robinson and John Edward Ellershaw in Palliative Care and Social Practice
