# Supplementary material for: ‘It’s given us the opportunity’: Patient and clinician experiences of serious illness conversations in the NHS acute setting – Results from a UK Qualitative Study
Source: Palliat Care Soc Pract. 2026 Jun 14;20:26323524261450637. doi: 10.1177/26323524261450637 (PMC13265982; doi:10.1177/26323524261450637)
Supplement: sj-docx-1-pcr-10.1177_26323524261450637 – Supplemental material for ‘It’s given us the opportunity’: Patient and clinician experiences of serious illness conversations in the NHS acute setting – Results from a UK Qualitative Study [file sj-docx-1-pcr-10.1177_26323524261450637.docx]

**Table 1**

Consolidated criteria for reporting qualitative studies (COREQ): 32-item checklist

| No | Item | Guide questions/description | Response |
| --- | --- | --- | --- |
| Domain 1: Research team and reflexivity | | | |
| Personal Characteristics | | | |
| 1. | Interviewer/facilitator | Which author/s conducted the interview or focus group? | TM (lead researcher) conducted all interviews. |
| 2. | Credentials | What were the researcher's credentials? *E.g. PhD, MD* | The first author of the manuscript undertook this research for a PhD thesis. She also has a Masters in Social Research Methodology. |
| 3. | Occupation | What was their occupation at the time of the study? | Research Assistant |
| 4. | Gender | Was the researcher male or female? | Female |
| 5. | Experience and training | What experience or training did the researcher have? | The first author of the manuscript undertook this research for a PhD thesis. She also has a Masters in Social Research Methodology. |
| Relationship with participants | | | |
| 6. | Relationship established | Was a relationship established prior to study commencement? | The researcher gained verbal consent to contact all potential participants and introduce the study. The researcher spoke to all potential participants to provide information and answer any questions. |
| 7. | Participant knowledge of the interviewer | What did the participants know about the researcher? e*.g. personal goals, reasons for doing the research* | Prior to providing consent to take part in the interviews, potential participants (patients and clinicians) were provided with information about the study and were encouraged to ask the research team questions about the study, including about the research team. |
| 8. | Interviewer characteristics | What characteristics were reported about the interviewer/facilitator? e.g. *Bias, assumptions, reasons and interests in the research topic* | The researcher was a research assistant in palliative and end of life care, conducting this research for a PhD. |
| Domain 2: study design | | | |
| Theoretical framework | | | |
| 9. | Methodological orientation and Theory | What methodological orientation was stated to underpin the study? *e.g. grounded theory, discourse analysis, ethnography, phenomenology, content analysis* | This qualitative study took a phenomenological approach, as the purpose was to explore the lived experience of participants. Reflexive Thematic Analysis was used within a constructionist paradigm. |
| Participant selection | | | |
| 10. | Sampling | How were participants selected? *e.g. purposive, convenience, consecutive, snowball* | Purposive sampling was used. Participants were selected based on inclusion/exclusion criteria, which are reported in the manuscript. |
| 11. | Method of approach | How were participants approached? e*.g. face-to-face, telephone, mail, email* | Patients were approached by their clinician; clinicians were approached during training. |
| 12. | Sample size | How many participants were in the study? | 14 patients and 9 clinicians. |
| 13. | Non-participation | How many people refused to participate or dropped out? Reasons? | 9 patients declined or died before interview; 4 clinicians declined. |
| Setting | | | |
| 14. | Setting of data collection | Where was the data collected? e*.g. home, clinic, workplace* | The participants took part in the interviews in their own home, either face to face with the interviewer, or over the telephone. |
| 15. | Presence of non-participants | Was anyone else present besides the participants and researchers? | Some patients had family members present during interviews. |
| 16. | Description of sample | What are the important characteristics of the sample? *e.g. demographic data, date* | This is presented within the manuscript. Details of the participants listed in tables 2 and 3. Detailed demographic tables provided for patients and clinicians, in tables 1 and 2. |
| Data collection | | | |
| 17. | Interview guide | Were questions, prompts, guides provided by the authors? Was it pilot tested? | The interviews were guided by topic guides developed for use in the study. |
| 18. | Repeat interviews | Were repeat interviews carried out? If yes, how many? | No |
| 19. | Audio/visual recording | Did the research use audio or visual recording to collect the data? | All interviews were audio recorded. |
| 20. | Field notes | Were field notes made during and/or after the interview or focus group? | During the interview the researcher made brief notes where appropriate and possible. Reflexive notes were made during familiarisation. |
| 21. | Duration | What was the duration of the interviews or focus group? | The interviews were around 1 hour, on average. |
| 22. | Data saturation | Was data saturation discussed? |  |
| 23. | Transcripts returned | Were transcripts returned to participants for comment and/or correction? | No |
| Domain 3: analysis and findings | | | |
| Data analysis |  |  |  |
| 24. | Number of data coders | How many data coders coded the data? | One – TM conducted coding and theme development. |
| 25. | Description of the coding tree | Did authors provide a description of the coding tree? | The themes generated have been described in the manuscript. |
| 26. | Derivation of themes | Were themes identified in advance or derived from the data? | Themes were derived inductively from the data. |
| 27. | Software | What software, if applicable, was used to manage the data? | Word and Excel were used. |
| 28. | Participant checking | Did participants provide feedback on the findings? | No |
| Reporting |  |  |  |
| 29. | Quotations presented | Were participant quotations presented to illustrate the themes / findings? Was each quotation identified? e*.g. participant number* | Yes, quotes with participant ID’s have been presented to illustrate themes. |
| 30. | Data and findings consistent | Was there consistency between the data presented and the findings? | Findings are supported by participant quotes. |
| 31. | Clarity of major themes | Were major themes clearly presented in the findings? | There three main themes are presented in the manuscript. |
| 32. | Clarity of minor themes | Is there a description of diverse cases or discussion of minor themes? | The themes have been presented so that the variation of responses across participants is highlighted. |
