# Supplementary material for: ‘It’s given us the opportunity’: Patient and clinician experiences of serious illness conversations in the NHS acute setting – Results from a UK Qualitative Study
Source: Palliat Care Soc Pract. 2026 Jun 14;20:26323524261450637. doi: 10.1177/26323524261450637 (PMC13265982; doi:10.1177/26323524261450637)
Supplement: sj-docx-2-pcr-10.1177_26323524261450637 – Supplemental material for ‘It’s given us the opportunity’: Patient and clinician experiences of serious illness conversations in the NHS acute setting – Results from a UK Qualitative Study [file sj-docx-2-pcr-10.1177_26323524261450637.docx]

Appendix 1: Patient and Clinician Interview Topic Guide and Prompts

**Patient Interview Topic Guide**

I am interested in understanding from your point of view, what it was like to be involved in the recent Serious Illness Conversation you had with [consultant], where you talked about your illness and plans for your future care. I would like you to describe in as much detail as possible, what you remember of the meeting, including who was present, what topics were discussed, how it made you feel, and how useful you think/feel the meeting has been. It would be helpful if you could also tell us something about your understanding of the purpose of the meeting, and your expectations following this meeting.

Potential prompts to elicit specific information (if required):

- 1. Did you understand the purpose of the meeting?
  2. Were you given time to prepare for the meeting?
  3. What were your expectations of the meeting?
  4. Had you thought about (and/or discussed) your future care with anyone prior to this meeting?
  5. Were your views sought and listened and responded to appropriately?
  6. How did talking about this topic make you feel?
  7. How useful do you think these conversations have been for you?
  8. What went well?
  9. What could have gone better?

**Clinician - Interview Topic Guide**

I am interested in understanding from your point of view what it is like to be involved in having serious illness conversations with your patients and using the serious illness conversation guide. Firstly, it would be helpful if you could describe in as much detail as possible, how patients were invited to a serious illness conversation, and what happens during these conversations, including your understanding of the purpose of these conversations. Secondly, we would like you to talk about how being part of the conversation made you feel, and how having this conversation may impact on your clinical practice and subsequent communication with patients.

Potential prompts to elicit specific information (if required):

1. What is your understanding of the purpose of having the serious illness conversation with your patients?
2. What are the ‘triggers’ or factors which have prompted you to arrange a serious illness conversation with your patients?
3. What are your expectations for your patients, as a result of having these serious illness conversations?
4. Did you feel that the patients understood the reason why the serious illness conversation had been arranged?
5. How did it feel for you to talk about this topic with the patient?
6. How do you think talking about this topic made the patient feel?
7. How useful do you think having these conversations has been for the patient?
8. How useful do you think having these conversations has been for you as a clinician?
9. What went well?
10. What could have gone better?
